# Prevalência de infecção por *Helicobacter pylori* em uma comunidade indígena em São Paulo e fatores associados: estudo transversal: Prevalence of *Helicobacter pylori* infection in an indigenous community in São Paulo and associated factors: cross-sectional study

**DOI:** 10.1590/1516-3180.2016.0114091216

**Published:** 2017-04-20

**Authors:** Juliana Rejane da Silva Roque, Rodrigo Strehl Machado, Douglas Rodrigues, Patrícia Rech, Elisabete Kawakami

**Affiliations:** I MD, MSc. Postgraduate Student, Discipline of Pediatric Gastroenterology, Department of Pediatrics, Escola Paulista de Medicina - Universidade Federal de São Paulo (EPM-Unifesp), São Paulo (SP), Brazil.; II MD, PhD. Clinical Instructor, Discipline of Pediatric Gastroenterology, Department of Pediatrics, Escola Paulista de Medicina - Universidade Federal de São Paulo (EPM-Unifesp), São Paulo (SP), Brazil.; III MD, PhD. Clinical Instructor, Department of Preventive Medicine, Escola Paulista de Medicina - Universidade Federal de São Paulo (EPM-Unifesp), São Paulo (SP), Brazil.; IV MSc. Postgraduate Student, Department of Preventive Medicine, Escola Paulista de Medicina - Universidade Federal de São Paulo (EPM-Unifesp), São Paulo (SP), Brazil.; V MD, PhD. Full Professor, Discipline of Pediatric Gastroenterology, Department of Pediatrics, Escola Paulista de Medicina - Universidade Federal de São Paulo (EPM-Unifesp), São Paulo (SP), Brazil.

**Keywords:** Prevalence, Helicobacter pylori, Child, Risk factors, Protective factors

## Abstract

**CONTEXT AND OBJECTIVE:**

The prevalence of *Helicobacter pylori* infection is unevenly distributed among different populations. The aim here was to evaluate the factors associated with *Helicobacter pylori* infection among children up to five years of age living in a high-risk community.

**DESIGN AND SETTING:**

Cross-sectional study in an indigenous community of Guarani *Mbya* ethnicity, Tekoa Ytu and Tekoa Pyau villages, Jaraguá district, city of São Paulo (SP), Brazil.

**METHODS:**

74 children aged 0.4 to 4.9 years (mean 2.9 ± 1.3 years; median 3.1), and 145 family members (86 siblings, 43 mothers and 16 fathers) were evaluated for *Helicobacter pylori* infection using the validated ^13^C-urea breath test. Clinical and demographic data were collected.

**RESULTS:**

The prevalence was 8.3% among children aged 1-2 years and reached 64.3% among those aged 4-5 years (P = 0.018; overall 31.1%). The prevalence was 76.7% among siblings and 89.8% among parents. There was a negative association with previous use of antibiotics in multivariate analysis adjusted for age (odds ratio, OR: 0.07; 95% confidence interval, CI: 0.01 to 0.66; P = 0.02). The prevalence was higher among males (OR: 1.55), and was associated with maternal infection (OR: 1.81), infection of both parents (OR: 1.5), vomiting (OR: 1.28), intestinal parasitosis (OR: 2.25), previous hospitalization (OR: 0.69) and breastfeeding (OR: 1.87).

**CONCLUSIONS:**

The prevalence was high among subjects older than three years of age, thus suggesting that the incidence of infection was higher over the first three years of life. Previous use of antibiotics was inversely associated with current *Helicobacter pylori* infection.

## RESUMO

**CONTEXTO E OBJETIVO:**

A prevalência da infecção pelo *Helicobacter pylori* é desigualmente distribuída entre diferentes populações. O objetivo foi avaliar fatores associados à infecção pelo *Helicobacter pylori* em crianças de até cinco anos de idade morando numa comunidade de alto risco.

**TIPO DE ESTUDO E LOCAL:**

Estudo transversal na comunidade indígena da etnia Guarani *Mbya* das aldeias Tekoa Ytu e Tekoa Pyau do distrito de Jaraguá da cidade de São Paulo (SP), Brasil.

**MÉTODOS:**

74 crianças de 0,4 a 4,9 anos (média 2,9 ± 1,3 anos; mediana 3,1), e 145 familiares (86 irmãos, 43 mães e 16 pais) foram avaliadas quanto à infecção pelo *Helicobacter pylori* com o teste respiratório com ureia ^13^C validado. Dados clínicos e demográficos foram coletados.

**RESULTADOS:**

Prevalência foi 8,3% entre 1-2 anos, atingindo 64,3% entre 4-5 anos (P = 0,018; 31,1% globalmente). Em irmãos, foi 76,7% e 89,8% nos pais. Observou-se associação negativa com uso prévio de antibiótico em análise multivariada ajustada para idade (*odds ratio*, OR: 0,07; intervalo de confiança, IC: 95%: 0,01-0,66; P = 0,02). A prevalência foi maior no sexo masculino (OR: 1,55), e foi associada a infecção materna (OR: 1,81), infecção de ambos os pais (OR: 1,5), vômitos (OR: 1,28), parasitose intestinal (OR: 2,25), hospitalização prévia (OR: 0,69) e aleitamento materno (OR: 1,87).

**CONCLUSÕES:**

A prevalência foi alta entre os indivíduos com mais de três anos de idade, o que sugere incidência maior nos três primeiros anos de vida. Uso prévio de antibióticos foi inversamente associado a infecção atual por *Helicobacter pylori*.

## INTRODUCTION

Although *Helicobacter pylori* infection is ubiquitous among humans, its prevalence is unevenly distributed among different populations. In developed countries, the incidence of this infection has declined sharply over the past few decades. Recently, prevalence of under 10% among children has been reported. In Brazil, the prevalence of *Helicobacter pylori* remains high among deprived urban settlements, rural communities and indigenous communities. Occurrences of *Helicobacter pylori* are probably due to poor sanitation, allied with poverty.^1^^,^^2^^,^^3^^,^^4^^,^^5^^,^^6^

Recently, we reported that there was high prevalence (73.5%) of this infection among children in six indigenous communities in the Amazon Forest.^6^ However, there is no study evaluating indigenous communities located in urban areas.

The infection is contracted primarily during childhood, with low incidence among adults. Although the precise mechanism of transmission is largely unknown, there is evidence that person-to-person transmission within households plays a role. Furthermore, in rural areas, transmission may also occur through contaminated food and water, and through close contact with non-maternal caregivers.^7^

*Helicobacter pylori* infection is associated with long-term morbidity (peptic ulcer disease, gastric cancer and gastric MALT lymphoma), but children may also be affected.^8^ Recently, we demonstrated that *Helicobacter pylori* eradication plays a role in treating chronic immune thrombocytopenic purpura in children.^9^ Nowadays, the burden of *Helicobacter pylori* infection is mostly borne by developing countries and specific high-risk groups. Studies conducted in such populations allow better knowledge of risk factors, especially if the study targets young children, since most new infections occur at that age.

## OBJECTIVE

The present study aimed to evaluate factors associated with *Helicobacter pylori* infection among children up to five years of age in an urban indigenous community of Guarani ethnicity in the city of São Paulo, Brazil.

## METHODS

### Subjects

Children up to five years of age living in an indigenous community of Guarani *Mbya* ethnicity based in the northwestern region of the city of São Paulo were eligible for inclusion. The total population was estimated as 636 people (demographic census of 2010), with 137 children under the age of 5 years. The community does not have any sewage system, running water or regular electricity supply. Also, the housing is precarious and most of the population lives in houses without toilets or showers. Consequently, most of the children defecate directly on the ground.

The community does not have anywhere for hunting or fishing, and the soil is unsuitable for growing crops. Therefore, the community raises money by selling crafts. Lastly, they have plenty of animals (cats, raccoons, monkeys and, especially, dogs).

### Study protocol

All the children up to five years of age and their family members living in the same dwelling (parents and siblings) were invited to participate in the study. Parents who were willing to participate were asked to answer a questionnaire that asked about breastfeeding, dummy/pacifier use, bottle-feeding, bed-sharing, number of household members, episodes of vomiting and/or diarrhea, use of antibiotics over the previous month and previous admissions to hospital. Parents were asked about the use of antibiotics over the previous month among index subjects (children up to five years of age). As the mothers understood the Portuguese language, the interviews were conducted in Portuguese by the principal researcher, always with help from a health agent from the community.

Index subjects and their family members (parents and siblings) performed a ^13^C-urea breath test so that cases of *Helicobacter pylori* infection could be diagnosed. In addition, all parents were requested to provide a stool sample from the index subject for stool and ova examination, as described below.

### ^13^C-urea breath test

After the participants had fasted for four hours, breath samples were collected through one-way valves connected to aluminum bags. Face masks were used to collect samples from children who were unable to blow voluntarily. Samples were collected at baseline and 30 minutes after ingestion of 50 mg of ^13^C-urea diluted in 100 ml of orange juice without added sugar. 

The breath samples were analyzed using an infrared isotope analyzer (IRIS; Wagner Analysen Technik, Bremen, Germany). Delta over baseline (DOB) greater than 4‰ was regarded as positive, as had previously been validated locally among children younger than six years of age (sensitivity 93.3%, with 95% confidence interval, CI: 86.8%-99.7%; specificity 96.2%, with 95% CI: 93.6%-98.8).^10^

### Stool ova and parasite test

Every caregiver was given a stool collector (PARATEST brand, Diagnostek) and was instructed to collect freshly evacuated stools. The samples were processed by means of the Hoffman method, with a subsequent search for eggs and cysts using optical microscopy.

### Study design and statistics

This was a cross-sectional analysis. The study included all the indigenous children with all their family members living in the same dwelling, and there was no sampling procedure. All indigenous children up to five years of age and their families were invited to participate in the study. Villages were visited three times a week by the principal investigator and a health worker.

Quantitative variables were described in terms of their means, medians and standard deviations, while categorical variables were described in terms of their proportions. Infection rate variation according to age group was tested by means of the chi-square test for linear trend. Otherwise, associations were assessed using the chi-square test or Fisher’s exact test. A binary logistic model was used to assess the association between *Helicobacter pylori* infection and selected variables. The independent variables were the following: sex of the child, vomiting, intestinal parasitosis, previous antibiotic use, previous hospitalization and breastfeeding, as well as infected mother and infected father. Independent variables that were found to be associated with the primary outcome (*Helicobacter pylori* infection in the child, i.e. the dependent variable) at a P-level of less than 0.25 were included in a multivariate logistic model. The fit of the multiple regression was assessed in accordance with the Hosmer-Lemeshow test, and P-values < 0.05 indicated a good fit. P-values < 0.05 were considered statistically significant. The statistical packages SPSS for Windows 17.0 and Stata for Windows version 10.0 were used for statistical analysis.

### Ethical considerations

The leaders of the community were given explanations about the study protocol, and they approved it. Moreover, every single subject and their respective legal guardian gave signed consent (assent for children over eight years of age). This study was approved by the Research Ethics Committee of the Paulista School of Medicine, Federal University of São Paulo, by the Ethics Committee of the Municipal Health Department of São Paulo and by the National Council for Research Ethics (Comissão Nacional de Ética em Pesquisa, CONEP).

## RESULTS

A total of 74 children (33 males and 41 females; age range ­0.4-4.9 years; median age 3.1; and mean age 2.9 ± 1.3) and 145 family members were evaluated. None of the parents actively refused to participate, but 63 of the 137 children (46%) did not show up on the scheduled date. None of these 63 children adhered to the study protocol, despite the various calls made personally in their homes. There was no compliance with the study protocol among these 63 children, in spite of multiple requests made by the study staff.

We do not believe there were any systematic differences between those who participated and those who did not. The family members comprised 86 siblings (46 males and 40 females; age range 5.1 - 16.8 years; mean age 10.9 ± 8.3 years; and median 9.0 years), 43 mothers (age range 15.7-56.4 years; mean 36.1 ± 28.8; and median 30.0) and 16 fathers (age range 22.4-54.9 years; mean 38.6 ± 23.0; and median 27.4) (Table 1).

**Table 1:**
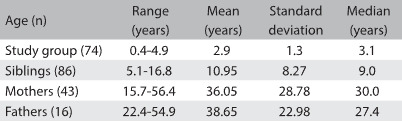
Descriptive statistics on the ages of the study population

The prevalence of *Helicobacter pylori* among the 74 children was 31.1% (23/74), with a significant direct relationship with age (P = 0.018), such that the prevalence ranged from 8.3% at the ages of 1-2 years to 64.3% at the ages of 4-5 years (Table 2). Positive stool and ova tests were not associated with *Helicobacter pylori* infection, given that nearly all the samples were positive (94.1%, 48/51) and 45.8% of them showed multiple infestation. *Endolimax nana* was the parasite most frequently detected concurrently (47.9%), followed by *Entamoeba coli* (35.4%), *Giardia lamblia* (31.3%) and *Ascaris lumbricoides* (31.3%).

**Table 2:**
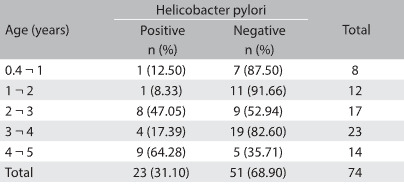
Prevalence of *Helicobacter pylori* infection among 74 children younger than 5 years of age

Fisher’s exact test: P = 0.018 (association test for linear trend).

The following variables were not associated with cases of *Helicobacter pylori* infection (P > 0.05): infected mother (odds ratio, OR: 1.81; 95% CI: 0.33 to 9.72), male gender (OR: 1.55; 95% CI: 0.58 to 4.19), maternal and paternal infection together (OR: 1.5; 95% CI: 0.27 to 8.29), vomiting (OR: 1.28; 95% CI: 0.41 to 4.03), intestinal parasitosis (OR: 2.25; 95% CI: 0.45 to 11.36), previous hospitalization (OR: 0.69; 95% CI: 0.22 to 2.2) and breastfeeding (OR: 1.87; 95% CI: 0.2 to 17.75) (Tables 3 and 4). On the other hand, previous use of antibiotics was inversely associated with *Helicobacter pylori* infection (OR: 0.07; 95% CI: 0.01 to 0.66).

**Table 3:**
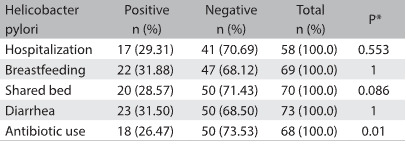
*Helicobacter pylori* infection and associated factors among 74 children younger than 5 years of age

*Fisher’s exact test.

**Table 4:**
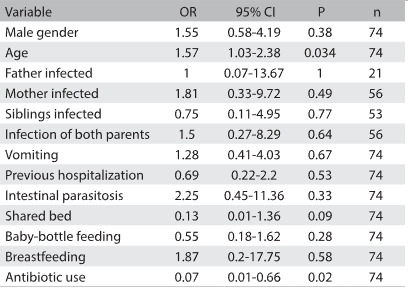
Factors relating to *Helicobacter pylori* infection among 74 children younger than 5 years of age

OR = odds ratio; CI = confidence interval.

In the multivariate analysis, both age (OR: 1.67; 95% CI: 1.05 to 2.67; P = 0.030) and previous use of antibiotics remained significantly associated with the outcome.

## DISCUSSION

In this urban community with high prevalence of infectious parasitic diseases, we found that nearly two-thirds of the children had become infected with *Helicobacter pylori* by the age of five years. This high prevalence rate at such a young age highlights the precarious living conditions in this community, with poor sanitation. Comorbidities such as infectious diarrhea were observed in 100% of the children, and intestinal parasites, in almost all of the children examined. In the same city (São Paulo), among children with low socioeconomic status, we previously reported that the seroprevalence of *Helicobacter pylori* infection among children aged 2-4 years was 20.8%, and among those aged 4-6 years, 25%.^5^ Furthermore, lower prevalence was found among adolescents (10-16 years of age) in a public school in the city of São Paulo (40.7%).^11^ It is clear that this urban indigenous community presented higher prevalence of this infection. The age group at highest risk of contracting the infection was the group younger than three years of age. At this age, around half of the children were infected. This high rate of early acquisition of infection needs to be taken into consideration in designing strategies to prevent infection in high-risk communities.^12^

In a prospective study, Rowland et al. described a baseline prevalence of 8.6%, and an incidence of 7.6% (20/262); 95% (19/20) of the new infections occurred in children younger than five years of age, with a maximum incidence rate at 2-3 years (5.05/100 person-years).^13^ On the other hand, in a high-risk population (indigenous children), 60% of the children aged 2-3 years were already infected and the prevalence of infection increased until 4-5 years of age (80.6%), and then increased slightly again at a later age (8-9 years; 85.3%).^6^ Thus, both high and low-prevalence populations seem to present the highest incidence in younger children.

We chose to evaluate children younger than five years of age in a high-risk population because that would be the age group with maximum incidence. Although there was a direct relationship between prevalence and age, children in their fourth year presented lower prevalence (17.39%). It can be hypothesized that spontaneous elimination of *Helicobacter pylori* (*H. pylori)* infection may have occurred in some children up to that age, as was shown by Klein et al., in a cohort of Peruvian children. These authors found that the prevalence among 18-month infants was lower than among 6-month infants (reduction from 71.4% to 47.9%), and they hypothesized that in young infants, *H. pylori* infection may be a reversible process, due to a particular immune response.^14^ A longitudinal study might elucidate whether spontaneous elimination of the infection plays any significant role in high-risk populations.

Spontaneous clearance in children has been observed in several studies,^14^^,^^15^^,^^16^ and there is the possibility that exposure to antibiotics might contribute towards spontaneous elimination of infection particularly in children.^16^^,^^17^ However, only a few studies have investigated the association between antibiotic use and infection by *H. pylori* among children aged less than four years,^17^^,^^18^ i.e. the period during which antibiotics are widely used and the incidence of *H. pylori* infection is relatively high.^19^ The present study supports the hypothesis that exposure to antibiotics aimed at other infections may be responsible for eradication of *H. pylori* infection in children. According to the Pasitos cohort study, spontaneous elimination of *H. pylori* infection was significantly related to incidental antibiotic exposure, but this only explained a small proportion of the spontaneous clearance rate.^20^

In the present study, it was unclear whether infection in children exposed to antibiotics was eradicated. Because the study group formed part of a small homogeneous indigenous village community, we believe that antibiotic use was not just an epiphenomenon, i.e. a sign of different access to health care. Serological tests might provide further information, since serum samples would remain positive long after eradication, but the lack of sensitivity of these tests in children precluded their inclusion in the present study protocol.^21^

Intrafamilial transmission of *H. pylori* infection could not be evaluated because of loss of parents and siblings from the sample. Only a few parents participated in the study (43 mothers and 16 fathers), which hampered proper assessment of intrafamily transmission. However, the prevalence of *H. pylori* was high among family members, and the chances that children would be infected were slightly greater if the mother was infected (OR: 1.81) or if both parents were infected (OR: 1.5), although these results were not statistically significant. Our results differed from those reported by Weyermann et al., in which maternal infection was the only strong and significant factor (OR: 13.0; 95% CI: 3.0 to 55.2) in an urban population.^22^ Transmission of pathogens between people in the same family is common, especially among children younger than five years of age. Contamination can occur through gastro-oral, oral or oral-fecal-oral transmission, and diarrhea, vomiting or regurgitation may facilitate it.^23^ The study population had high hospitalization rates and almost all participants had had an episode of acute gastroenteritis, which may have favored transmission of *H. pylori*.

Recently, a systematic review supported the notion that breastfeeding has a protective role against *H. pylori* infection among children living in economically less developed settings.^24^ In the present study, however, breastfeeding was not protective against *H. pylori* infection. Similarly, Rowland et al. reported that there was no difference in the rate or duration of breastfeeding between infected and uninfected children. However, in another study, in a high-prevalence country (77% at 12 months of age), exclusive breastfeeding was associated with reduced risk.^25^

The prevalence of *H. pylori* infection was high over the first five years of life but incidence was higher in the first three years of life, which indicates the importance of implementing immediate public health and sanitation measures. These preventive measures are the key to reducing the prevalence of infection over the initial years of life. In these areas of high risk of infection, the data suggest that antibiotic use is inversely associated with *H. pylori* infection.
